# Effects of the two carvone enantiomers on soil enzymes involved in the C, P, and N cycles

**DOI:** 10.1186/2241-5793-21-7

**Published:** 2014-05-20

**Authors:** Efimia M Papatheodorou, Chysanthi Margariti, Despoina Vokou

**Affiliations:** Department of Ecology, Aristotle University of Thessaloniki, Thessaloniki, UP Box 119 54124, Greece

**Keywords:** Aromatic plants, Carvone, Enantiomers, Essential oil, Dehydrogenase, Phospho-monoesterase, Urease

## Abstract

**Background:**

Essential oils exert stimulatory or inhibitory effects on the size and activity of the soil microbial communities. Given that microbial biomass is the main source of soil enzymes, in this study, we examined how *R*-(-)- and *S*-(+)-carvone affect the activity of dehydrogenase, urease, and alkaline phospho-monoesterase, and the overall microbial activity, as expressed by soil respiration. Enzymatic and microbial activities were recorded every week, for a period of four weeks, during which the two carvone enantiomers were added twice, with a two-week interval, into soil samples. For all dependent variables, we analysed the deviations of the experimental from control values.

**Results:**

Treatment *per se* had a significant effect only on urease. Its activity was inhibited in the *S*-carvone samples, while it was enhanced or inhibited, depending on the time of incubation, in the *R*-carvone ones. The activity of alkaline phospho-monoesterase was not affected by *S*-carvone, but it increased with *R*-carvone. Soil respiration markedly increased in presence of the two carvones with highest values being recorded in the *R*-carvone samples. None of the temporal patterns of the three enzymes’ activity followed the pattern of soil respiration.

**Conclusions:**

The significant treatment by time interactions for the activities of all three enzymes indicates that responses are not consistent over time; this suggests differently functioning or structured microbial communities. Given their differing effects on soil enzymes, these compounds and the aromatic plants bearing them could find use in sustainable agriculture for the control of soil enzymes and, hence, the soil processes that they are associated with.

## Background

Aromatic plants are very common in the Mediterranean environment. Their essential oils are biologically very active inducing both stimulatory and inhibitory effects on plants, herbivores and microorganisms [1, 2]. Falling leaves of aromatic plants may still contain large amounts of essential oils [3]; these are expected to affect soil processes and organisms.

Some essential oil constituents are found to exhibit strong antifungal activity regarding both growth and sporulation, whereas others to stimulate sporulation of some fungi [4]. Several studies show that essential oils have always stimulatory effects on soil respiration with some of these effects being immediate, whereas others occurring after a time lag [5–8]. This is accompanied by an increase of the size of soil bacterial populations but also shifts in the composition of soil microbial communities [8, 9]. Although the effects of essential oils on size, composition and activity of soil microbial communities are rather extensively studied, their effects on soil processes mediated by specific enzymes remain largely unknown.

In this study, we use *R*-(-)- and *S*-(+)-carvone, two monoterpenoid ketones, which occur in the essential oils of various aromatic plants but are major constituents of *Mentha spicata*[10] and *Carum carvi*[11], respectively. We explore their effect on the activity of soil enzymes that have microbes as their main source [12, 13] and which play crucial roles in the C, N, and P cycles: dehydrogenase, urease, and alkaline phospho-monoesterase, respectively. In parallel, we explore their effect on the overall activity of the soil microbial community, as expressed by soil respiration. We follow the microbial and enzymatic activities over a period of a month and we compare their temporal patterns. A similarity would allow us to interpret the changes observed in microbial activity as associated primarily to the changes in activity of the specific enzyme(s). We also examine how similar are the responses induced by *R*-(-)- and *S*-(+)-carvone. These compounds are enantiomers, i.e. structurally identical molecules but mirror images of each other rotating plane-polarized light in opposite directions (+/-). Enantiomers may differ in their reaction with other substances that are also enantiomers. Given this and the fact that many of the molecules in living beings are of this type, there may be marked differences in the effect of the two compounds [14, 15].

## Results

**Figure 1:**
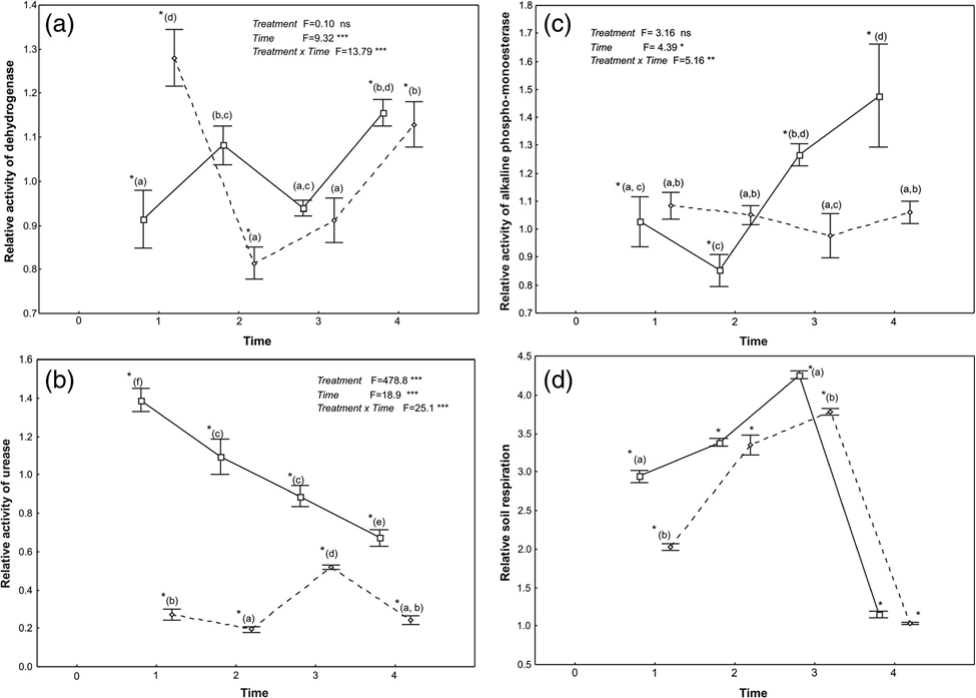
**Effects of** ***R*** **-(-)- (continuous line) and** ***S*** **-(+)-carvone (dotted line) on relative activity (mean ± standard error). (a)** dehydrogenase, **(b)** urease, **(c)** alkaline phospho-monoesterase and **(d)** relative soil respiration, during the incubation period lasting four weeks. Carvones were added at times 0 and 2. Letters correspond to significant differences due to the combined effect of treatment (compound) with time for enzymatic activities and to the treatment effect for soil respiration; asterisks denote significant differences between treatments and corresponding controls, at each sampling point, separately (χ^2^ test) (ns = non significant differences, * = *p* < 0.05, ** =*p* < 0.01, *** = *p* < 0.001).

Soil respiration and alkaline phospho-monoesterase, dehydrogenase, and urease activities over the four-week experimental period are presented in Table 1 as absolute values; in Figure 1, they are presented in relative terms, indicating how much they deviate from the control. Factorial ANOVA showed no effect of treatment on relative dehydrogenase activity, but a highly significant temporal effect as well as a combined effect of treatment x time (Figure 1a). Both applications (in the beginning and in the middle of the experiment) of *R-*carvone led to a decrease of dehydrogenase activity a week later, which, nonetheless, was significantly lower than in the control only after the first time that it was applied. An increase followed, two weeks later, which, nonetheless, was significant only after the second time that it was applied. The response of dehydrogenase activity to *S-*carvone was different: it increased a week after the first application, dropped dramatically two weeks later, and increased again two weeks after the second application. It is to be noted, that although the response of dehydrogenase activity was compound-specific the first time that the two carvones were supplied, it was similar when they were supplied for a second time.

**Table 1:** **Mean values (±standard errors) of enzymatic activities and soil respiration in** ***R*** **- and** ***S*** **-carvone treated samples**

Week	Enzymatic activity						Soil respiration (mg CO_2_ d^-1^)	
	Dehydrogenase (μg g^-1^ 24 hr^-1^)		Alkaline phospho-monoesterase (mg kg^-1^ hr^-1^)		Urease (mg kg^-1^ 2 hr^-1^)			
	***R***-(-) carvone	***S***-(+) carvone	***R***-(-) carvone	***S***-(+) carvone	***R***-(-) carvone	***S***-(+) carvone	***R***-(-) carvone	***S***-(+) carvone
1^st^	9.4 ± 0.71	13.2 ± 0.69	444.1 ± 40.2	468.6 ± 21.4	129.7 ± 5.94	25.2 ± 2.84	6.5 ± 0.18	5.2 ± 0.72
2^nd^	15.7 ± 0.66	11.8 ± 0.56	390.5 ± 27.2	481.2 ± 16.1	102.1 ± 8.87	18.1 ± 1.37	2.5 ± 0.04	2.5 ± 0.10
3^rd^	11.2 ± 0.35	10.4 ± 0.60	577.7 ± 19.4	445.6 ± 38.6	81.9 ± 5.12	47.9 ± 1.07	4.7 ± 0.06	4.1 ± 0.05
4^th^	12.4 ± 0.34	12.1 ± 0.59	544.3 ± 71.6	390.9 ± 15.2	62.5 ± 4.09	22.5 ± 2.13	1.0 ± 0.04	0.9 ± 0.02

Significant treatment, time, and time x treatment effects were detected for the relative activity of urease (Figure 1b). During the whole incubation period, it was far higher in the *R*-carvone-enriched samples (2- to 5-fold) than in the *S*-carvone ones. Moreover, urease activity in the *S*-carvone-enriched samples was far lower than in control samples throughout the incubation period and did not differ among sampling times, except a week after the second application, when it showed a temporary increase. In contrast, in the *R*-carvone enriched samples, urease activity increased and remained at high levels even the second week after the first application, but it decreased and continued to fall after the second time that this compound was applied.

Alkaline phospho-monoesterase relative activity exhibited no response to treatment but was affected by time, and treatment x time (Figure 1c). Supply of *S-*carvone had no effect whatsoever, either in the beginning or in the middle of the experiment. On the contrary, both applications of *R-*carvone brought about a significant increase of the enzymatic activity that was larger after the second application.

There was a treatment effect on the relative activity of the soil microbial community (Figure 1d). In presence of either of the two compounds, soil respiration became remarkably higher than in the control samples, even three or four times, with *R*-carvone inducing more pronounced responses both in the beginning (F = 95.71, *p* < 0.001) and in the middle (F = 50.53, *p* < 0.001) of the experiment. Enhancement was less prominent but lasting the first time that they were supplied, whereas it almost disappeared a week later, the second time. Despite these quantitative differences, the temporal pattern of changes in the presence of the two compounds was almost identical.

## Discussion

The prominent feature in the response of the soil microbial community to the two carvones was the remarkable increase of respiration, which dropped dramatically, almost to the levels of the control, two weeks after the second addition.

It is known that essential oil constituents are quite rapidly decomposed. Vokou & Margaris [7] estimated that soil bacteria consume them in about 15 days, whereas Lehmann *et al.*[16] reported that the two carvones are consumed in 12 days by indigenous microorganisms of river waters that also use them as growth media. The respiration response that we observed is consistent with a consumption time of about 12 to 15 days and suggests that decomposition of the newly added substrates is the dominant soil process following addition of these compounds.

Parallel to respiration increase, incorporating essential oils or their individual constituents into the soil result into increases of the size of soil bacterial populations [5–7] and into major shifts in the composition of bacterial communities [8, 9], inducing in parallel both inhibitory and stimulatory effects on different bacteria or fungi [4, 8]. Lehmann *et al*. [16] also found changes in the microbial community structure of river waters induced by *R*- and *S*-carvone, with the community reverting back to that persisting before the carvones supply. Changes of the microbial community structure towards bacteria tolerant to essential oil constituents or able to use them as substrates of growth, and reversibility of the resulting community after their removal were also reported by Chalkos [9]. These are indications of a dynamic system and suggest that the soil microbial community may not have been the same at the two time points that the carvones were supplied, what could explain the differences in the enzymatic responses observed in our experiments.

Throughout the experimental period, respiration changes followed exactly the same pattern in the presence of either of the two carvone enantiomers: whenever added into the soil, they enhanced soil respiration, *R*-carvone to a greater extent. This similarity of respiration changes did not hold true for any of the three enzymatic activities examined, with differences in the patterns induced by the two compounds ranging from small to dramatic. As none of the temporal patterns of the three enzymes’ activity was similar to that of soil respiration, we can conclude that the processes in which they are involved are not primarily responsible for the overall pattern of microbial respiration.

From the three enzymes studied, only the response of urease was clearly enantioselective. However, for all three enzymes, a significant treatment x time effect was recorded indicating the dependence of the enzymes’ response on the specific microbial community structure and/or function prevailing at the time; more pronounced responses to the second addition could imply an acclimation of specific microbes to the compound added.

Changes in the relative activity of dehydrogenase, involved primarily in the carbon cycle and being a fundamental part of the enzyme system of all living microorganisms [17], did not match those of soil respiration, but a number of interesting features were observed. The temporal pattern of soil respiration was antiparallel to that of dehydrogenase activity in the *R*-carvone treated samples. More specifically, the two patterns were negatively related (*p* < 0.01, *r*^2^ = 0.42, b = -0.55), but there was nothing similar in the *S*-carvone treated samples. Dehydrogenase activity is related to the aeration status of the soil: it is negatively correlated to the redox potential and air filled porosity [18]. We could argue, therefore, that, despite opening the soil containers every week, the more intense respiration in the *R*-carvone samples compared to the *S*-carvone ones resulted to high oxygen consumption and hence oxygen limitation, leading in turn to predominance of facultative anaerobes in the metabolic processes [19].

Regarding urease, we found an effect of treatment on its relative activity. This enantioselectivity was expressed in the following way: *S*-carvone exhibited a clearly inhibitory effect, whereas *R*-carvone had a varied effect depending on the time it was supplied: enhancement at first, inhibition later. Trying to minimize N losses, Patra *et al.*[20] tested chemicals that retard urea hydrolysis (urease inhibitors) or nitrification. They found that *Mentha spicata* essential oil retarded nitrification, but it was not clear whether it also inhibited urea hydrolysis. In our experiments, we found that *R*-(-)-carvone, which is the major constituent of *Mentha spicata,* inhibited urease activity and, hence, urea hydrolysis, only after the second supply; also, that its enantiomer *S*-(+)-carvone was much more inhibitory and effective throughout the experimental period. The enhancement of urease activity that was detected in the *R*-carvone treated samples after the first addition may have resulted to higher amounts of NH_4_^+^ in the soil, which would in turn increase soil pH favouring ammonia volatization [21]. But this we did not examine.

Although alkaline phospho-monoesterase is an enzyme exclusively produced by microbes [22], it showed no response to the addition of *S*-carvone, but it showed to *R*-carvone. To our knowledge, this is the first study exploring the effects of essential oil constituents on soil phosphatases. Hence, no comparison to literature data is possible. However, other studies have shown the activity of this enzyme to remain unaffected by various treatments like sylvicultural ones [23] or to show abnormal responses, as is the case of the spectacular enhancement (10-fold) of activity in soil receiving herbicides [24].

## Conclusions

The patterns of enzymatic activity, as influenced by the two carvones, did not match the pattern of overall metabolic activity and suggest intervention of complicated mechanisms, not understood so far. Differing effects of enantiomers have been reported for a number of processes but not for soil enzymatic activity, whereas responses of enzymatic activity after repeated interventions have not been examined either. The ability of these compounds to stimulate or inhibit soil enzymatic activity could find novel applications in organic or sustainable agriculture. Depending on the soil process that farmers would like to enhance or inhibit, they could apply the compound inducing the desired effect or with plant bearing it. The potential of these natural products to control the activity of soil enzymes and, hence, soil processes needs to be further explored.

## Methods

### Soil and chemicals

We used air-dried and sieved (2 mm sieve) soil from the top layer of an area in the farm of the Aristotle University of Thessaloniki that was left in fallow for many years. The soil consisted of 32% clay, 56% silt, 12% sand, 1.5% organic matter and 7.5% CaCO_3_. Soil pH was 8.2 and had cation exchange capacity 28.6 meq 100 g^-1^[25]. The two carvones were commercially supplied [Sigma Chemical Co (St. Louis, MO, USA)]. Purity is 96% for *S*-(+)-carvone and 98% for *R*-(-)-carvone.

### Experiments

The methods that we used, the quantities of the compounds and the time that they were added were according to a scheme developed and tested earlier [5], which was repeatedly applied afterwards [6–8]. More specifically, air-dried soil samples (75 g) mixed with water (25 ml) were put in 500-ml hermetically closed containers. The compounds under investigation were added a week later. This is because soil responses to the presence of essential oils or of their individual constituents immediately after the first wetting are not always clear [6] with soil requiring about a week to stabilize. The two compounds were added twice, with a two-week interval, at a quantity of 0.05 ml each time. A repeated application can reveal whether responses are the same, irrespective of the time that a substance is supplied, or time-specific, and hence, depending on the features of the prevailing microbial community. Samples were incubated at 27 ± 2°C, in the dark. The experiments lasted four weeks with starting point (time 0) being the time when the carvones were first supplied; first measurements were taken a week later. Containers were opened at sampling times, once every week. Each time, a small quantity of water (1 ml) was added to adjust for possible losses.

For the estimation of soil enzymatic activity, we used two series of 32 containers, one for each carvone isomer; half of the containers corresponded to the treated and half to the control samples. For each enzyme, the activity was recorded following a destructive sampling: four containers with treated soil and another four with control samples were chosen at random and removed on a weekly basis for a total of four weeks. At each sampling time, all other flasks were opened and closed again for oxygen to be replenished and water to be added so that all samples were treated in the same way.

For the estimation of soil respiration, we used four control and four treated samples per compound. For this analysis, a beaker with KOH (10 ml) was put in the containers to absorb the CO_2_ that was released during incubation; this was afterwards determined by titration for both treated and control samples. Same as for enzymatic activity, CO_2_ was measured on a weekly basis, but sampling was not destructive: new beakers with KOH replaced the previous ones in the same containers.

### Enzymatic activity

The activities of urease and alkaline phospho-monoesterase were determined following the methods described by Tabatabai [12]. Of the phospho-monoesterases, we monitored the activity of the alkaline phospho-monoesterase due to the alkaline pH of the soil that we used. Enzymatic activity was assayed at optimal pH value for each enzyme. Dehydrogenase activity was determined by reduction of 2,3,5-triphenyltetrazolium chloride (TTC) to formazan, according to Casida *et al.*[26]. Enzymatic activity is expressed as μg TRF g^-1^ 24 hr^-1^, mg *p*-nitrophenol kg^-1^ hr^-1^ and mg NH_3_-N kg^-1^ 2 hr^-1^, for dehydrogenase, phospho-monoesterase and urease, respectively.

### Statistical analysis

All experiments could not run concurrently because of the limited capacity of the incubator. For this reason, there were different control samples for the two treatments (*R*- and *S*-carvone). To compare the effects of the two compounds, we estimated the deviations of the experimental values from the control ones. We thus estimated relative respiration as the respiration of the treatment divided by that of the corresponding control, and similarly, relative enzymatic activity (for each of the three enzymes examined) as the enzymatic activity of the treatment divided by that of the corresponding control. We examined by *t*-test analysis whether there were significant differences among the controls of each treatment for each of the variables examined. In all cases, no significance differences were detected, so a common control value was used. For each sampling time and treatment, a *X*^2^-test was applied to check whether the estimated values deviated from the control.

To analyse the effect of treatment (compound examined) and time on relative enzymatic activity, a Factorial ANOVA followed by Fischer post-hoc comparisons was performed. Data were transformed whenever the assumptions of ANOVA (normality, equality of variances, independence of means and variances) were not met. More specifically, data for urease and alkaline phophatase were log and 1/square root transformed, respectively. For soil respiration, as sampling was not destructive, a repeated measures ANOVA was applied, and differences among treatments for each sampling point were detected. The statistical analyses were conducted using Statistica 7 for Windows (StatSoft, Tulsa, USA).

## Abbreviations

TTC: 2,3,5-triphenyltetrazolium chloride.
